# HER2 Negative Mammary Paget's Disease or In Situ Melanoma? A Case Report and Review of the Literature

**DOI:** 10.1155/2023/1101130

**Published:** 2023-05-23

**Authors:** Luana-Andreea Boșoteanu, Mariana Așchie, Cristian Ionuţ Orǎșanu, Mădălina Boșoteanu

**Affiliations:** ^1^Department of Dermatology, “Elias” Emergency University Hospital, Bucharest, Romania; ^2^Institute of Doctoral Studies, Doctoral School of Medicine, “Ovidius” University of Constanta, Constanta, Romania; ^3^Clinical Service of Pathology, “Saint Andrew” Emergency County Hospital, Constanta, Romania; ^4^Department of Pathology, Faculty of Medicine, “Ovidius” University of Constanta, Romania; ^5^Department VIII-Medical Sciences, Academy of Romanian Scientists, Bucharest, Romania

## Abstract

Mammary Paget's disease (MPD) is a rare histological condition, accounting for 1-4% of female breast cancers, which may appear either independently (1.4-13% of the cases), or in association with an in situ or invasive ductal carcinoma (approximately 90% of the cases). The purpose of this article is to highlight the histopathological challenges related to the microscopical polymorphism of this disease and the utmost importance of immunohistochemistry in the thorough process of Paget's disease differential diagnosis. Moreover, the primary objective of this review of literature was to corroborate the existing data concerning the potential peculiar immunohistochemical profile that mammary Paget's disease might express. We report the case of a 44-year-old female patient, histopathologically diagnosed with HER2-negative MPD accompanying an invasive mammary carcinoma. The histopathological and immunohistochemical approach is derived from the exigency of excluding the possibility of synchronous tumors—a mammary invasive carcinoma, accompanied by another component with MPD phenotypic mimicry. The unexpected negative HER2 reaction is conducted to a primary focus on excluding a malignant melanoma in situ. The absence of MelanA and S100 immunoexpression and lack of pigmentation and clinical aspects infirmed it. Bowen's disease was invalidated by its rare presentation in the breast cutaneous tissue and the absence of individual risk factors suggestive of an existing immunosuppressive status. In the case of similar morphoimmunohistochemical aspects, significant expression of Ki-67 signals MPD, an immunoreactivity that helped distinguish the cellular population from Toker cells. The great similarity of MPD with other benign and malignant cutaneous tumors might determine delay or misdiagnosis. Thus, the utmost importance of immunohistochemistry is reflected in its prognostic significance and geared towards extending the therapeutic arsenal.

## 1. Introduction

Mammary Paget's disease (MPD) was first described in 1874 by Sir James Paget [1], who reported a unilateral chronic eczematous dermatitis of the nipple-areola complex, representing the extension through continuity of an intraductal carcinoma of the breast. This rare histological condition, accounting for 1-4% of female breast cancers, may appear either independently (1.4-13% of the cases) or in association with an in situ or invasive ductal carcinoma (approximately 90% of the cases) [2].

The main clinical aspects comprise relatively well-defined oval-shaped red plaques covered by scaly patches that bring to sight a wet suppurative surface after detachment; the lesions develop induration and infiltration during their evolution [3]. Regional lymphatic dissemination is more frequently encountered when MPD is associated with a palpable tumoral mass in the breast [4]. Patients might be asymptomatic or accuse pruritus, a burning sensation, local discharge, hemorrhages, ulcerations, or nipple invagination [5].

A prompt and accurate diagnosis dictates the therapeutic arsenal and, subsequently, the prognosis. Five-year postoperative survival rates vary from 92% (absence of a clinically detectable mass) to 38% (palpable breast tumor), supplementarily darkened by the regional lymphadenopathies [6]. The purpose of this article is to highlight the histopathological challenges related to the microscopical polymorphism of this disease and the utmost importance of immunohistochemistry (IHC) in the thorough process of Paget's disease differential diagnosis.

## 2. Case Presentation

### 2.1. Clinical History

**Figure 1:**
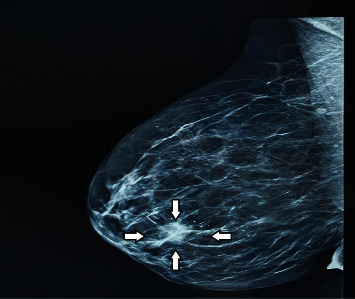
Mammographic aspect of the right breast tumoral mass—BIRADS score of 5.

We present the case of a 44-year-old female patient presented to the General Surgery Department of “Saint Andrew” County Clinical Emergency Hospital of Constanţa with a right mammary mass, previously identified on a unilateral digital mammography (Figure 1). This paraclinical investigation found a heterogeneous mass in the right breast; the irregular opacity of high intensity presented fine spicules and measured 18/16 mm, associating microcalcification foci, with apparent ductal distribution, characteristics highly suggestive for malignancy, and a BIRADS score of 5.

### 2.2. Paraclinical Investigations

Furthermore, the clinical examination revealed cutaneous retraction and a firm painless lump in the superior-intern quadrant of the right breast, with a diameter of approximately 8/6 cm. A 2/1-centimeter pseudo-tumoral mass was palpated in the right axilla. The craniocerebral CT scan identified right frontal arteriovenous malformation (Spetzler-Martin, grade II) and left maxillary sinusitis. Supplementarily, the thoracic computed tomography confirmed the presence of the right mammary mass and described a right axillary expansive lesion, probably with a fibrotic substrate, without other suspect elements. Therefore, the patient underwent a right Madden modified mastectomy with right axillary lymphadenectomy and drainage.

### 2.3. Pathology

The macroscopical evaluation identified mammary parenchyma with dimensions of 6.5/4.5/1.5 cm with a poorly defined nodular translucent lesion measuring 1.4/1.6/1.7 cm, of medium consistency. Retracted nipple-areola complex, with existent mobility on the subjacent planes, reddish aspect, and small erosions were observed.

Microscopically, the paraffin-embedded tissue obtained after the processing of the specimen, corresponding to the described nodule, displayed histopathological lesions of invasive mammary carcinoma (NST), moderate grade of histological malignancy (G2), Nottingham score of 7 (tumor tubule formation—3 points; nuclear pleomorphism—2 points; mitotic activity—2 points), with perineural and lymphovascular invasion. Moreover, in situ ductal carcinoma lesions of solid-, flat-, and comedo-type, with intermediate nuclear grading, were associated. Stromal fibrohyalinization was accompanied by calcifications and chronic granulomatous inflammatory infiltrate in an active form. Two out of 14 lymph nodes sampled through the dissection of axillary tissue revealed breast carcinoma metastasis, with the maximal diameter of the tumoral deposit of 18 mm and extracapsular extension of 4 mm in size.

The histopathological diagnosis of an invasive mammary carcinoma (NST), with axillary lymph node metastases (pT1 N1a M0 LVI+ PNI+), was established.

**Figure 2:**
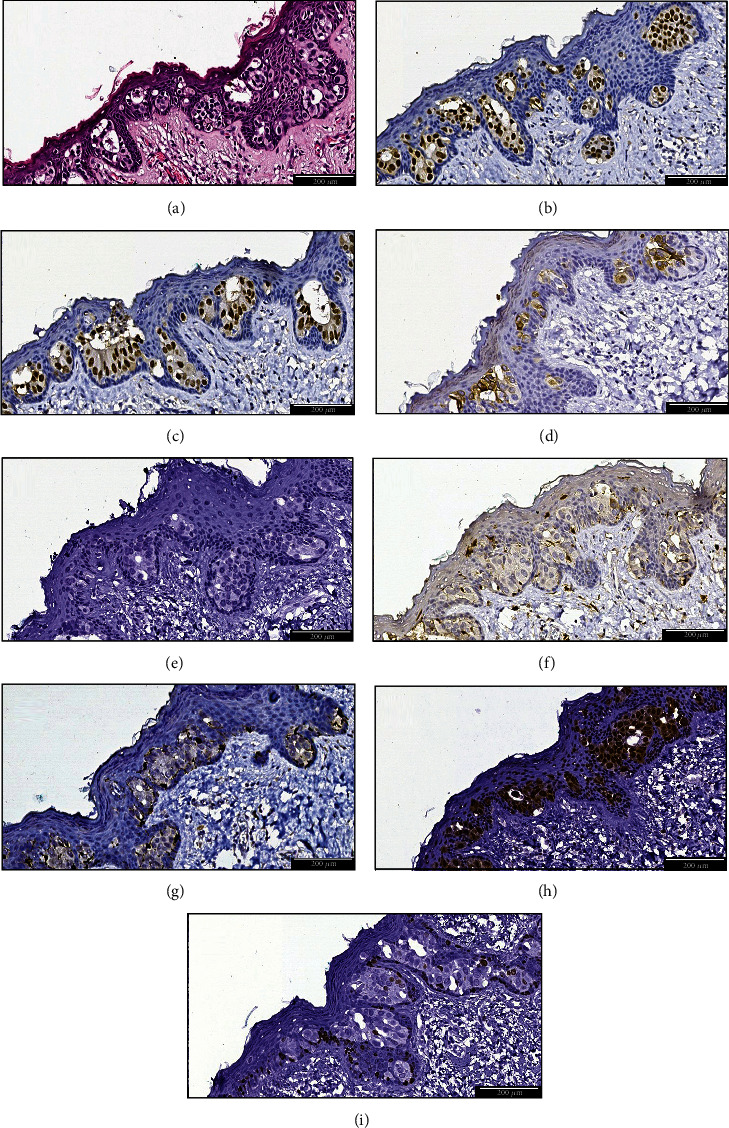
Microscopic aspect and immunohistochemical stainings of the intraepithelial tumoral proliferation: (a) HE ×200; (b) ER ×200; (c) PR ×200; (d) CK7 ×200; (e) HER2 ×200; (f) S100 ×200; (g) MelanA ×200; (h) GATA3 ×200; (i) Ki-67 ×200.

Furthermore, the mammary papilla revealed the presence of medium-large, polygonal cellular population, with abundant pale eosinophilic cytoplasm and unique/multiple nuclei with conspicuous nucleoli, at the transepidermal level. The neoplastic proliferation mainly respected a nested, occasionally tubular, pattern, associating isolated tumoral cells (Figure 2(a)). A minimal invasion (<1 mm) in the superficial dermis was identified; the converged histopathological elements oriented the diagnosis towards mammary Paget's disease.

Subsequently, immunohistochemical testing was recommended. This evaluation was performed with a panel of 8 antibodies from Biocare Medical, using the manual method, according to the current protocols, on 5 *μ*m-thick sections of formalin-fixed, paraffin-embedded tumoral tissue blocks (Table 1). The invasive component of breast carcinoma has been included in the luminal B molecular subtype. The intraepithelial neoplastic component revealed positive immunostaining for estrogen receptor (90% of the malignant cellular population; Figure 2(b)), progesterone receptor (40% of the malignant cellular population; Figure 2(c)), CK7 (Figure 2(d)). Diversely, HER2 had a negative reaction (score 0) in the intraepidermal neoplastic population (Figure 2(e)), fact supported by the in situ hybridization molecular tests that displayed a nonamplified HER2 status. Negative reactions for S100 (Figure 2(f)) and MelanA/MART1 (Figure 2(g)), with positive internal control—dermal and intraepidermal melanocytes—were detected. Moreover, GATA3 displayed a positive reaction in the intraepidermal tumoral cells (Figure 2(h)). Ki-67 revealed a positive nuclear reaction of moderate intensity in 75% (hotspot) of the intraepithelial neoplastic proliferation (Figure 2(i)). Thus, the exposed immunophenotype was compatible with the diagnosis of Paget's disease of the breast, HER2 negative. MPD was histopathologically diagnosed and immunohistochemically evaluated, revealing a distinct profile.

## 3. Discussion

Laborious MPD diagnosis of certainty may occur, due to the similar clinical aspect with other dermatological entities. Paget's disease of the breast shall be differentiated from melanoma in situ (MIS), atopic dermatitis, psoriasis, Bowen's disease, squamous metaplasia of the lactiferous ducts (SMOLD)/Zuska's disease, papillary adenoma of the nipple, nevoid hyperkeratosis of the nipple and areola (NHNA), and pagetoid dyskeratosis [7]. The use of IHC stainings, such as cytokeratin, CERBb2 oncoprotein, and EMA (epithelial membrane antigen), is requested to assemble the differential diagnosis correctly and exhaustively (Table 2).

The great similarity of this pathological entity with other benign and malignant conditions of the skin might determine delay or misdiagnosis of MPD. Furthermore, the characteristic negative reaction for HER2 of the presented tumor was conducted to an enhanced evaluation, followed by a thorough differential diagnosis. In the present case, the latter mainly assessed entities with a characteristic negative HER2 immunohistochemical reaction, such as melanoma in situ, Bowen's disease, Toker cells, and pagetoid dyskeratosis. The purpose of this meticulous diagnostic approach was motivated by the exigency of excluding the possibility of two synchronous tumors—a mammary invasive carcinoma, accompanied by one of the previously mentioned diseases.

Typically, the immunoprofile for MPD described in the literature comprises positivity for CK7 (>90%) [8], HER2 (80-100%) [9], CAM5.2 (70-100%) [10], CEA (5 out of 7 cases) [11], ER (10-40%) [12], and PR (28.6%) [13]. Among these, HER2 is described as the immunohistochemical stain most likely to be positive in Paget's disease [14]. The present literature data states that the vast majority of MPD displays HER2 overexpression, via immunohistochemical techniques [9]. According to a recent publication, the key role of GATA3 as a marker of MPD has been proved by its positive reaction in 95% of complex Paget's disease cases, including but not limited to those that comprise CK7 negativity [15]. In the current case, Paget's disease, as well as the invasive carcinoma component of the tumor, was hormone receptor positive and HER2 negative. In this context, the unexpected negative reaction dictated a precise differential diagnosis.

In our reported case, the primary concern was distinguishing the potential diagnosis of MPD from that of a malignant melanoma in situ (MIS). In the case of MIS, the pathology reports describe groups of atypical melanocytes, confined to the epidermis, without dermal invasion, hence not breeching the basement membrane, while Paget's cells are distributed more diffusely [16]. Furthermore, MIS does not present lumen formation nor mucin positivity, while the microscopic aspect of MPD indicates the possibility that atypical cells form lumens or are mucin positive. On a clinical and histological level, rare cases of Paget's disease are pigmented, but melanin can, however, be present in both processes [17].

Considering the prominent phenotypic mimicry of the latter with various malignant neoplasms, the assessment of immunohistochemical markers is essential. Among them, S-100 exhibits the most sensitive profile for melanocytic lesions and generates a positive immunoreaction in approximately 100% of MIS but only displays positivity in 0-26% of MPD cases [18]. A common marker for diagnosing malignant melanoma in situ is represented by MelanA/MART1, the monoclonal antibody that we decided to examine to clarify the differential diagnosis. The reaction obtained after assessing the tissue sample was negative, thus excluding the suspicion of MIS.

Classic diagnostic instruments used for melanoma are, among others, MelanA and HMB45, whose mechanisms are distinct. A recent study stated that these markers are not always reliable and sufficient for the distinction between MPD and melanoma, a more thorough examination of multiple melanocytic markers being suggested [19].

The otherwise high sensitivity of the S100 protein—through its presence/absence—aids the diagnostic process. Several cases included in a comparative pathological study revealed positive immunoexpression of this protein in the Paget cells, highlighting the importance of data corroboration for extracting the veridic role of each marker in the accurate diagnosis of pigmentary cutaneous lesions [20].

Supplementarily, the positive reaction for GATA3 in our case guided towards the diagnosis of MPD.

Moreover, there is no strict histologic distinction between Paget cells and melanoma cells, hence the necessity of immunohistochemical staining, but certain features may guide towards the correct diagnosis [21]. In case of invasive melanoma suspicion, its characteristic cells are usually objectified transepidermally, with associated pagetoid spread and occasional dermal invasion. In contrast, Paget's cells display a different localization, suprajacent to the basal keratinocytic layer of the epidermis, presenting ductal formation, without dermal expression [22].

As it was stated by Rao et al., the combined presence of neoplastic ductal cells and atypical melanocytes in a unique sample might occur due to the existence of a single mammary tumor of breast origin, undergoing bidirectional dedifferentiation [23]. Furthermore, the melanocytic population objectified in the epidermal layer was one of the prominent elements that raised the differential diagnosis challenge. The slight pigmentation of the lesion was postulated as being a result of Paget's cells releasing chemoattractant that augments the number of dendritic melanocytes and melanophages [23].

Secondly, the previously presented immunohistochemical tumoral profile implied eliminating the suspected possibility of Bowen's disease, a squamous cell carcinoma in situ [14]. Bowen's disease reveals histopatologically individual cell keratinization and multinucleation. Moreover, IHC identifies positivity for CK5/6 [17], in this case, associated with negative reactions to CK7, CEA, and HER2. The occurrence of a relatively typical case of Bowen's disease of the nipple—published in the medical literature—arising in a male patient affected by the acquired immune deficiency syndrome (AIDS) supports the role of immunosuppression in its pathogenesis [24]. Therefore, the rare presentation of this disease in the breast cutaneous tissue, the absence of individual risk factors suggestive for an immunosuppressive status of the patient [24], and the positive immunoreaction for CK7 and GATA3 in our case excluded this hypothesis.

Thirdly, the numerous and atypical microscopical presence of Toker cells (TCs) imposes distinction from Paget's cells. The first represents epithelial cells, located along the basal epidermal layer of the nipple, usually without cytologic atypia [25]. TCs are present with roundish and scant chromatin nuclei. They are found incidentally and show a positive reaction for EMA, CAM5.2, and cytokeratin 7^2525^ but negativity for HER2/neu oncoprotein, fact that served as a supplementary criterion of difficulty in the differential diagnosis of the present case. Contrastively, Paget's disease cells (PDCs) are malignant glandular epithelial cells, and atypia is observed; they have abundant, pale-staining cytoplasm which might include mucin secretion vacuoles and pleomorphic and heterochromatic nuclei. Park et al. indicated that significant expression of Ki-67 is consistent with MPD [26]; therefore, this immunoreactivity helped us distinguish the cellular population from Toker cells.

Finally, pagetoid dyskeratosis represents an incidental finding that has been differently defined by various authors. Santos-Briz et al. conducted a study that determined the criteria necessary for a keratinocyte to be considered a Pagetoid dyskeratosis cell as shown in the following: a larger size than usual, with pale eosinophilic cytoplasm and pycnotic nucleus surrounded by a clear halo [27]. Immunohistochemically, Pagetoid dyskeratosis cells displayed negativity for EMA, CK7, CEA, and HER2 [28]. The atypical immunophenotype of our case imposed the differential diagnosis, aided by the microscopic aspect of the cellular proliferation and the additional CK7 and GATA3 immunopositivity.

Factors of MPD negative prognosis comprise the existence of a palpable breast mass, the histological tumoral type, lymph node enlargement, and age younger than 60 years [29].

Regarding the prognostic factors, in the absence of invasive carcinoma, HER2 positivity does not influence treatment since Paget's disease is an in situ carcinoma [29]. Breast carcinomas with the previously mentioned positive immunophenotype are linked to stronger invasiveness and poor prognosis. The 10-year survival of Paget's disease of the breast associated with invasive ductal carcinoma was lower than that of Paget's disease of the breast without invasive ductal carcinoma (49% vs. 64%), but the variety was attributed to HER2 overexpression [30]. Luminal A and luminal B types present higher 3-year disease-free survival than the one encountered in triple-negative and HER2 overexpression [31]. HER2-positive rate in breast carcinomas with MPD is higher than that in breast carcinomas without MPD; the ER/PR positive percentage in breast carcinomas with MPD is lower than that in breast carcinomas without MPD [32].

MPD often displays negative immunoprofiles as regards to estrogen (ER) and progesterone receptors (PR) because the underlying carcinomas incline to be highly aggressive. In our case, the tumoral profile distinctively showed overexpression of ER, conjoined with HER2 negativity, a type of association only encountered in 6% of the cases assessed in a study conducted by Nakai et al. [33].

## 4. Conclusion

Mammary Paget's disease is a rare but well-defined pathological entity of the breast, which may provoke to diagnostic pitfalls, due to the occasionally polymorphic immunohistochemical profile and its ambiguous variety of clinical expressions. We reported the present case to highlight the importance of the correct application of a widened panel of immunohistochemical antibodies in all similar clinical contexts. Due to its particular and rare immunoprofile, originating from the negative HER2 reaction, a meticulous histopathological assessment was mandatory. The fundamental elaboration of a complex differential diagnosis was based on excluding the possibility of two synchronous tumors. The inconsistent prognosis and evolution of the multiple pathologies integrated in the differential diagnosis spectrum of MPD imply exemplary medical knowledge and multidisciplinary participation, geared towards minimizing the final diagnosis challenge.

## Figures and Tables

**Table 1:** The immunohistochemical panel.

Antibody	Biocare Medical clone
Estrogen receptor	SP1
Progesterone receptor	SP2
Cytokeratin 7	BC1
c-erbB-2/*HER2*	EP3; EP1045Y
S100	15E2E2-4C4.3
MelanA/MART1 pan melanoma CK2	M2-7C10+M2-9E3+T31
GATA3	L50-823
Ki-67	SP6

**Table 2:** The differential diagnosis of Paget's disease of the breast [7].

Condition	Characteristics
Atopic dermatitis	(i) Benign (ii) Immune system disturbance, epidermal barrier dysregulation (iii) Pathology: hyperkeratosis, dyskeratosis, psoriasiform hyperplasia, patent intercellular edema

Allergic contact dermatitis	(i) Benign (ii) T-cell-mediated immune response, with delayed-type hypersensitivity response (iii) Pathology: hyperkeratosis and parakeratosis, eosinophilic spongiosis and microvesicles

Psoriasis	(i) Benign (ii) Hyperproliferation of the keratinocytes (iii) Dysregulation of the immune system (iv) Auspitz's sign (v) Pathology: hyperplasia of the epidermis, Munro microabscesses, parakeratosis

Squamous metaplasia of the lactiferous ducts (SMOLD)/Zuska's disease	(i) Benign (ii) Blockage of the lactiferous ducts by a keratin plug results in duct rupture and keratin debris penetrating the stroma (iii) No lymphadenopathy (iv) Pathology: squamous epithelium extended beyond the normal transition line within the duct orifice

Nipple adenoma/papillary adenoma of the nipple	(i) Benign (ii) Adenomas in the large lactiferous ducts of the nipple (iii) Pathology: epithelial hyperplasia with luminal obliteration or intraductal papillary projections, intraductal necrosis, pseudo-invasion of the ducts conferred by the distorting fibrosis

Nevoid hyperkeratosis of the nipple and areola (NHNA)	(i) Benign (ii) Frequent in premenopausal women (iii) Pathology: epidermal acanthosis, hyperkeratosis, and papillomatosis

Bowen's disease	(i) Benign can evolve into malignant (ii) Dysplasia and pleomorphism of the keratinocytes, with hyperchromatic nuclei, without dermal infiltration

Pagetoid dyskeratosis	(i) Benign (ii) Cells with a larger size than usual, with abundant pale eosinophilic cytoplasm and pycnotic nucleus surrounded by a clear halo and lacking cytologic atypia (iii) Negativity for EMA, CK7, CEA, and HER2

Inflammatory breast cancer	(i) Malignant (ii) Lymphatic blockage caused by cancer cells in the cutaneous tissue of the breast (iii) Usually associated with lymphadenopathy (iv) Pathology: tumor cells invasion of the dermal lymphatics

In situ malignant melanoma	(i) Malignant (ii) Involvement of MAPK/ERK pathway, N-RAS, or BRAF (iii) ABCDE algorithm (iv) Pathology: groups of intraepidermal atypical melanocytes

## Data Availability

The data supporting the conclusions of this study are found within the article and by consulting the works cited.
